# The Melanin-Concentrating Hormone as an Integrative Peptide Driving Motivated Behaviors

**DOI:** 10.3389/fnsys.2017.00032

**Published:** 2017-05-29

**Authors:** Giovanne B. Diniz, Jackson C. Bittencourt

**Affiliations:** ^1^Laboratory of Chemical Neuroanatomy, Department of Anatomy, Institute of Biomedical Sciences, University of São PauloSão Paulo, Brazil; ^2^Center for Neuroscience and Behavior, Institute of Psychology, University of São PauloSão Paulo, Brazil

**Keywords:** MCH, hypothalamus, energy homeostasis, feeding behavior, reward, foraging, metabolism

## Abstract

The melanin-concentrating hormone (MCH) is an important peptide implicated in the control of motivated behaviors. History, however, made this peptide first known for its participation in the control of skin pigmentation, from which its name derives. In addition to this peripheral role, MCH is strongly implicated in motivated behaviors, such as feeding, drinking, mating and, more recently, maternal behavior. It is suggested that MCH acts as an integrative peptide, converging sensory information and contributing to a general arousal of the organism. In this review, we will discuss the various aspects of energy homeostasis to which MCH has been associated to, focusing on the different inputs that feed the MCH peptidergic system with information regarding the homeostatic status of the organism and the exogenous sensory information that drives this system, as well as the outputs that allow MCH to act over a wide range of homeostatic and behavioral controls, highlighting the available morphological and hodological aspects that underlie these integrative actions. Besides the well-described role of MCH in feeding behavior, a prime example of hypothalamic-mediated integration, we will also examine those functions in which the participation of MCH has not yet been extensively characterized, including sexual, maternal, and defensive behaviors. We also evaluated the available data on the distribution of MCH and its function in the context of animals in their natural environment. Finally, we briefly comment on the evidence for MCH acting as a coordinator between different modalities of motivated behaviors, highlighting the most pressing open questions that are open for investigations and that could provide us with important insights about hypothalamic-dependent homeostatic integration.

## The Melanin-Concentrating Hormone[S]

The existence of the MCH was postulated in the 1930s as a factor that induces pallor in the skin of amphibians and participates in these animals’ mechanism of background color adaptation (Hogben and Slome, 1935). It took almost 50 years, however, for MCH to be isolated for the first time from the chum salmon (*Oncorhynchus keta*) hypophysis, where it plays exactly its hypothesized role in skin pigmentation by triggering a concentration of melanin in melanophores that results in pallor (Kawauchi et al., 1983). Although the chronology of MCH discovery emphasized its role in skin pigmentation, it is now proposed that this function only emerged for the first time in the last universal common ancestor of teleosts and holoceans, millions of years after the appearance of MCH synthesis in chordates (Kawauchi and Baker, 2004).

It was later discovered that teleosts have two paralogs of MCH, termed MCH1 and MCH2 (Ono et al., 1988), that have different patterns of distribution in the CNS. MCH1 corresponds to the peptide isolated by Kawauchi et al. (1983) and is mostly associated with the control of skin pigmentation. Berman et al. (2009), working with the zebrafish (*Danio rerio*) hypothalamus, demonstrated that MCH1 immunoreactivity is largely impacted by background color, reflecting its role in skin pigmentation, while only MCH2 displays pronounced alterations in fasted animals. This correlation between hypothalamic MCH2 and the energy balance of the organism indicates that MCH may be linked to motivated behaviors and integrative functions at least since the divergence of tetrapods and ray-finned fishes at ∼440 million years ago (Amores et al., 2011).

Six years after the discovery of MCH1 in the salmon hypophysis, Nahon et al. (1989) and Vaughan et al. (1989) described for the first time the mammalian MCH coding gene and structure. Using rat samples, these researchers showed that there is a single mammalian MCH, with 19 amino acids and a cysteine bridge that confers it a cyclic structure, synthesized from a 165 amino acids precursor coded by the *Pmch* gene. This precursor also contains the sequence of two other peptides, NEI and NGE, and although some functions are now attributed to the former (for a review, see Bittencourt and Celis, 2008), the biological activity of the latter is largely unknown. Synteny, genomic structure, and sequence identity indicate that the mammalian MCH is an ortholog of teleost MCH2 (Berman et al., 2009) and shows remarkable conservation when compared to the single MCH of a cartilaginous fish, the scalloped hammerhead shark (*Sphyrna lewini*), with a single substitution at the C-Terminal (Mizusawa et al., 2012).

## Anatomical Aspects

**FIGURE 1:**
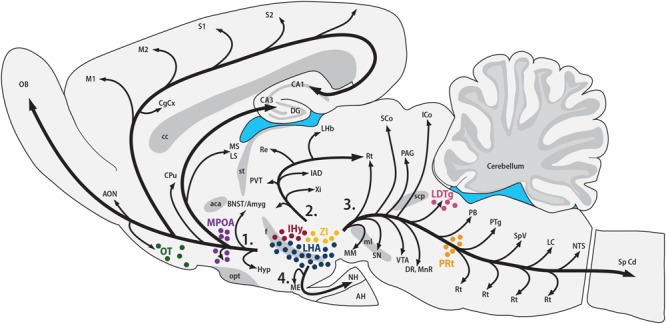
**MCH synthesis is found in discrete areas of the rat nervous system.** Sagittal representation of the rat central nervous system illustrating the main areas where MCH-ir neurons are found. The hypothalamic areas of synthesis in all animals are: the LHA (blue) and the IHy (red). Extra-hypothalamic areas are: the ZI (yellow), the OT (green) and the paramedian PnRt (orange). Exclusively in female rats, one extra-hypothalamic site can be found, in the laterodorsal tegmental nucleus (pink). During the lactation period, MCH-immunoreactive neurons can be detected in the medial preoptic area (purple). The four main pathways of MCH-ir fibers are also represented: 1 – ascending pathway; 2 – periventricular pathway; 3 – descending pathway; and 4 – hypophysaire pathway.

The synthesis of MCH shows two remarkably conserved features: most, if not all, MCH-immunoreactive (MCH-ir) neurons are found in the hypothalamus, and those neurons have widespread projections throughout the CNS. The first anatomical characterization of MCH was made in the male and female rat by Bittencourt et al. (1992). In this species, most MCH-ir neurons are found in the LHA and, to a lesser extent, in the AHA and PHA of the medial zone and in the dorsalmost area of the periventricular zone. A second group of rostral neurons is found in the IHy, previously known as rostromedial ZI (Bittencourt et al., 1992; Murray et al., 2000c), where MCH-ir neurons are intermingled with the DA neurons of the A13 group (Sita et al., 2003, 2007). Exclusively during the postpartum period, immunoreactivity to MCH can be detected in neurons of the MPOA and at the rostralmost aspect of the PVH, with an intensity that follows the progression of lactation and, with weaning, disappears (Knollema et al., 1992; Rondini et al., 2010). Outside the hypothalamus, MCH-ir neurons were described in the OT, ZI, and paramedian PnRt (Bittencourt et al., 1992) and in the LDTg of the female rat (Rondini et al., 2007). Little is known about the role played by MCH in these extra-hypothalamic areas. **Figure 1** summarizes the *loci* of MCH synthesis in the rat CNS and the main pathways formed by MCH-ir fibers.

**FIGURE 2:**
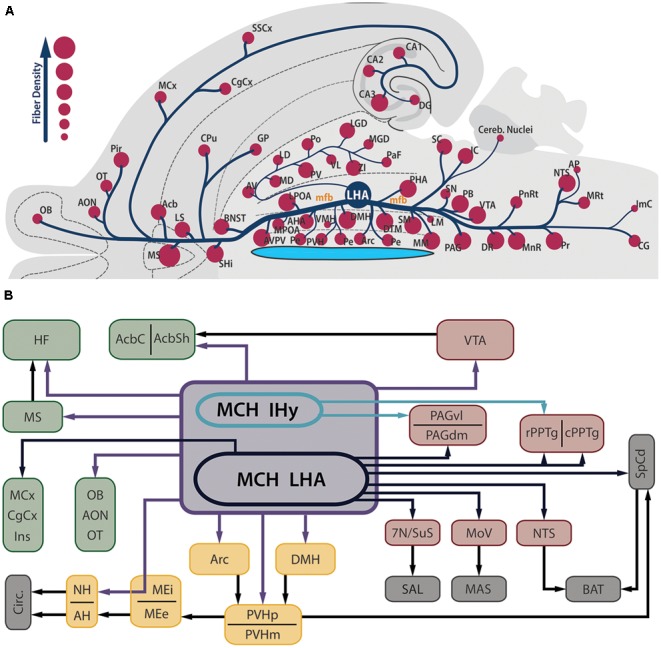
**MCH-immunoreactive fibers are profusely distributed in the neuroaxis and are found in several areas related to feeding behavior and energy homeostasis. (A)** Flatmap representation of the rat central nervous system illustrating the main areas where MCH-ir fibers can be found. The relative density of MCH-ir fibers in each area is indicated by the size of the circles. Observe that septal, hypothalamic and brainstem areas are densely innervated by MCH neurons, while cortical and thalamic areas display a smaller number of fibers. Due to the position of the LHA, most of the MCH-ir fibers leave this region intermingled to the mfb, with the periventricular pathway as a notable exception to this rule. **(B)** Schematic representation of the main targets of MCH-ir fibers in the rat CNS. Dark blue arrows represent projections originated from MCH-ir neurons located in the LHA, while green arrows indicate projections preferentially originated from the IHy. Purple arrows indicate projections originated from both areas or a lack of hodological studies to pinpoint the origin of such projections. Dark arrows indicate indirect connections relevant to this circuit. The main targets of MCH innervation can be arranged in three groups: telencephalic projections, in green; hypothalamic-hypophysaire projections, in yellow; and brainstem projections, in red. Downstream targets of MCH action are represented in gray.

Contrasting to the highly restricted pattern of MCH synthesis, MCH-ir fibers are found widespread throughout the whole CNS. Among the regions that contain fibers are olfactory areas, the septal nuclei, the basal nuclei, the HF, the neocortex, several diencephalic nuclei, the mesencephalic and PnRt, the PAG and all levels of the SpCd. The regions with the sparsest presence MCH-ir fibers are the cerebellum and some motor nuclei of the brainstem. In most of these areas, MCH-ir fibers are varicose and present many boutons *en passage* and boutons *terminaux*, suggesting an extensive synaptic activity (Bittencourt et al., 1992). **Figure 2A** is a visual representation of the MCH-ir fiber density in different areas of the rat CNS.

The distribution of MCH-ir cells has been studied in a wide range of animals, including agnathans (Mizuno, 1991; Bird et al., 2001), cartilaginous fish (*elasmobranchii*) (Vallarino et al., 1989; Mizusawa et al., 2012), teleosts (Naito et al., 1985; Batten and Baker, 1988; Powell and Baker, 1988; Bird and Baker, 1989; Minth et al., 1989; Gröneveld et al., 1995; Mancera and Fernandez-Llebrez, 1995; Amano et al., 2003; Huesa et al., 2005; Matsuda et al., 2009; Suzuki and Yamamoto, 2013), anurans (Andersen et al., 1986; Francis and Baker, 1995; Lázár et al., 2002), reptiles (Cardot et al., 1994), and birds (Cardot et al., 1998, 1999). In mammals, in addition to the rat (Bittencourt et al., 1992), MCH-ir cell bodies have been mapped in the mouse (Elias et al., 2001), Djungarian hamster (Khorooshi and Klingenspor, 2005), Syrian hamster (Vidal et al., 2005), tufted capuchin monkey (Bittencourt et al., 1998), humans (Elias et al., 2001), pigs (Chometton et al., 2014), sheep (Tillet et al., 1996), and cats (Torterolo et al., 2006). For a review of the anatomical aspects of MCH in mammals, see Bittencourt (2011).

Albeit there are some differences between these animals, the main *locus* of MCH synthesis in all of them is the hypothalamus or homologous structures, indicating that MCH-ir neurons reside this region at least since the emergence of the last vertebrate common ancestor. There is also a remarkable conservation in the widespread distribution of MCH-ir fibers. The hypothalamus, through its extensive projections, is a key structure for the execution of motivated behaviors, as understood as behavioral programs directed to exogenous factors that are executed by animals and result in a benefit to the survival of the organism or of its species (Swanson, 2005). This long phylogenetic history accounts, at least partially, for the numerous functions on which MCH has been suggested to play a role in, and may explain how MCH acquired an important integrative role in motivated behaviors centered on its hypothalamic localization supported by a widespread fiber distribution.

## Feeding Behavior

The most thoroughly described function of MCH is on the modulation of feeding behavior. The injection of MCH in the ventricle of rats (Qu et al., 1996; Rossi et al., 1997; Della-Zuana et al., 2002; Clegg et al., 2003; Duncan et al., 2005; Sakamaki et al., 2005) and mice (Gomori et al., 2003; Glick et al., 2009), as well as in the PVH (Rossi et al., 1997) or in the Acb of rats (Georgescu et al., 2005; Mul et al., 2011), provokes an increase in chow consumption followed by a gain of body weight and fat mass. Rossi et al. (1997), however, demonstrated that only acute injections of MCH can induce these effects, while chronic administration is unable to maintain it. Injections of MCH in the 3V also induce an increase in water and saccharin consumption in rats, independently of chow availability, indicating that MCH participates in hydric homeostasis processes (Clegg et al., 2003; Sakamaki et al., 2005; Furudono et al., 2006). This same treatment, however, increases alcohol consumption, suggesting that MCH participates in hedonistic aspects of this behavior as well (Duncan et al., 2005). Mice deficient in leptin synthesis [*Lep*^ob/ob^] with an obese phenotype display a higher expression of *Pmch* mRNA in the LHA when compared to WT mice, and this expression increases following fasting (Qu et al., 1996). MCH-overexpressing mice display higher chow consumption and increased body weight, as well as circulating levels of leptin and insulin resistance (Ludwig et al., 2001).

Data obtained from genetic models started broadening the roles associated to MCH. Shimada et al. (1998) report that *Pmch^-/-^* mice are leaner than *Pmch^+^* cohorts, but several evidence point to this leaner phenotype as the result of multiple physiological alterations. Not only *Pmch^-/-^* animals eat less, but they displayed increased oxygen consumption rates; their ability to overeat in response to starvation is not affected, although *Pmch^-/-^* animals have greater weight loss and higher mortality rate; and restricted food availability resulted in similar decreases in food intake between *Pmch^-/-^* and *Pmch^+^* mice, but deficient animals displayed increased weight loss. Another important evidence came with the work of Marsh et al. (2002), that reports that MCHR1-deficient mice have increased locomotor activity and energy expenditure that underlie an apparently contradictory hyperphagia observed in these animals. Finally, *Lep^ob/ob^ Pmch^-/-^* mice are leaner than *Lep^ob/ob^Pmch^+^*, but their food consumption is not decreased (Segal-Lieberman et al., 2003). This leaner phenotype is attributed to factors linked to energy expenditure, with higher motor activity and basal temperature in response to cold. Taken together, these results indicate that MCH acts on energy homeostasis through multiple pathways, including energy expenditure, locomotor activity and reward, while increases in ingestive behavior promoted by MCH are secondary in nature. In this section, we will review the multiple aspects of MCH action over nutritional homeostasis.

Although variations in dietary parameters are capable of modulating MCH neurons, with fasting increasing *Pmch* expression, variations in this expression may also underlie the preference of some animals for a specific diet. For example, Morganstern et al. (2010b) identified a subset of Sprague-Dawley rats that are prone to overconsume a high-fat diet (HFD) when compared to animals that show a baseline consumption of HFD. These authors demonstrated that animals that ingest a higher amount of HFD during a 5-day period (as measured by caloric intake) have increased levels of *Pmch* expression in the LHA when compared to animals that were kept in the same diet but ingested a lower number of calories. This difference persists even after the animals have been maintained in regular chow for weeks, suggesting that this intrinsic variation in *Pmch* levels may generate the preference of these animals for the higher fat content or increased palatability of the diet. This is extremely relevant because a similar mechanism may underlie human pathologies related to the excessive consumption of fat-rich foods. To explore alterations in the MCH peptidergic system in other models of natural or induced preference may generate important knowledge on the human physiology of ingestive behaviors (Barson et al., 2012).

### Inputs

**FIGURE 3:**
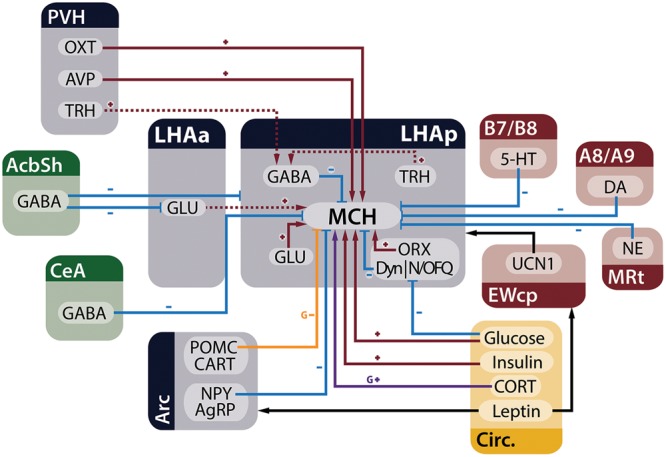
**MCH neurons receive several inputs relevant to the control of energy homeostasis.** Neurons from the telencephalon (green), hypothalamus (blue), and brainstem (red), in addition to circulating factors (yellow), are capable of modulating electrophysiological and genetic expression properties of MCH neurons in the LHA. Red arrows with plus signs represent depolarizing inputs, blue arrows with minus signs represent hyperpolarizing inputs, black arrows indicate relevant indirect connections to the circuit, the purple arrow represents increased expression of the *Pmch* gene and the orange arrow an input that decreases gene expression. Dashed lines indicate connections that have ample physiological support but lack morphological demonstration or may be composed of multiple polysynaptic elements. Some presynaptic modulatory inputs to GABA- and GLUergic neurons were omitted for clarity.

Melanin-concentrating hormone neurons are influenced by many inputs, including other neurons in the LHA, adjoining hypothalamic nuclei, telencephalic and brainstem areas, in addition to circulating factors that act as metabolic cues. **Figure 3** summarizes the main homeostatic-relevant inputs to MCH neurons.

#### Lateral Hypothalamic Area

The LHA is classically characterized by two populations of neurons, one that is MCH-ir (Bittencourt et al., 1992) and one that is ORX-ir (Peyron et al., 1998), both intermingled with GABAergic and GLUergic interneurons (Gao and van den Pol, 2001) who provide extensive input to MCH neurons through ionic and metabotropic channels (Gao and van den Pol, 2001; Huang and van den Pol, 2007). These two populations coexist in the LHA but are almost completely segregated, with a minimal number of neurons that co-synthesize these two peptides (Broberger et al., 1998; Elias et al., 1998; Peyron et al., 1998). There are two orexin peptides, ORX_A_ and ORX_B_, coded by the same gene (*Hcrt*) and involved in multiple functions related to homeostatic control and arousal (for recent reviews, see Mahler et al., 2014 and Sakurai, 2014). Both MCH and ORX neurons have profuse immunoreactive fibers in the LHA, contacting numerous neighboring cells (Bittencourt et al., 1992; Peyron et al., 1998). Although the high density of MCH-ir fibers close to MCH-ir cells could point to a recurrent effect of MCH on MCH cells, probably inhibitory due the nature of MCH neurons (Gao and van den Pol, 2001), there are no alterations in the electrophysiological properties of MCH neurons after MCH application in LHA slices (van den Pol et al., 2004).

**FIGURE 4:**
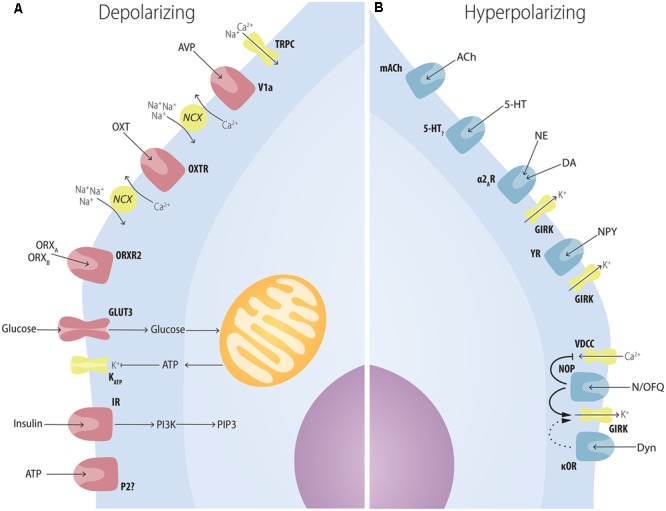
**Multiple factors have postsynaptic effects on MCH neurons. (A)** Several neuropeptides increase the excitability of MCH neurons through postsynaptic mechanisms. AVP depolarizes MCH neurons through NCXs and TRPC; OXT increases Na^+^ currents through NCXs; ATP in the extracellular medium increases MCH excitability through unknown mechanisms that may depend on P2?; orexins depolarize MCH neurons with special affinity for ORX_B_, suggesting an ORXR_2_-dependent pathway; glucose increases neuronal excitability in a mechanism similar to pancreatic β-cells: GLUT3 transporters increase the amount of glucose in the intracellular medium, boosting the amount of produced ATP, what blocks ATP-sensitive potassium channels (K_ATP_); insulin excites MCH neurons and activates PIP3-dependent pathways. **(B)** Several neuropeptides decrease the excitability of MCH neurons through postsynaptic mechanisms. ACh and 5-HT, two important elements of the central arousal system, inhibit MCH neurons; both NE and DA are capable of decreasing MCH excitability through the activation of the adrenoceptor type 2A and resulting activation of K^+^ currents through GIRK channels; NPY hyperpolarizes MCH neurons also through GIRK currents; opioids such as N/OFQ and Dyn hyperpolarize MCH neurons with convergent mechanisms through the blockage of VDCC and an increase in GIRK currents.

**FIGURE 5:**
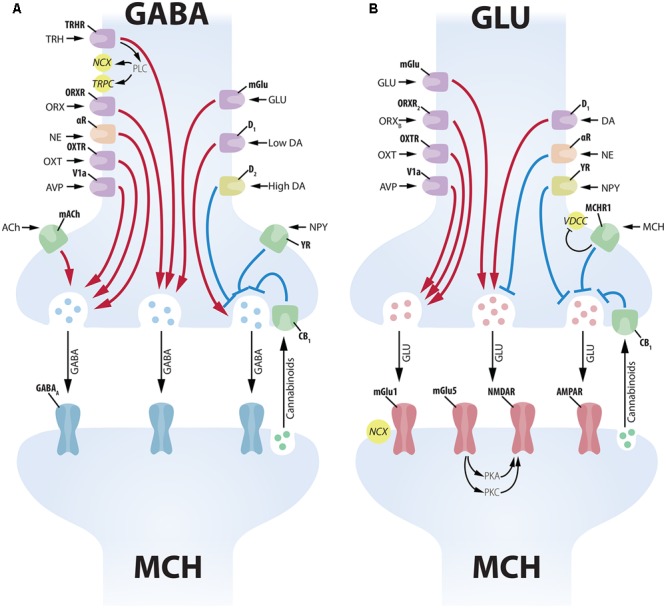
**Complex presynaptic modulation of MCH neurons. (A)** Several neuropeptides increase GABAergic input to MCH neurons acting on the soma of GABA neurons, such as: TRH, ORX, NE, OXT, AVP, GLU and low levels of DA. ACh increases GABAergic input to MCH neurons acting on axon terminals. High levels of DA, on the other hand, promote an inhibition of presynaptic GABAergic input through D_2_ receptors. NPY decreases inhibitory input acting on presynaptic terminals, while cannabinoids released by the MCH postsynaptic terminal decrease vesicle release (Huang et al., 2007). GABAergic input to MCH neurons is vehiculated mainly by GABA_A_ receptors. **(B)** The GLUergic input to MCH neurons is similarly under the control of numerous inputs. GLU, ORX, OXT, AVP, and DA act on the soma of GLU cells to increase excitatory input to MCH neurons. NE and NPY have the opposite effect, decreasing GLU input to MCH neurons. MCH has an inhibitory presynaptic effect through an MCHR1-dependent decrease in Ca^2+^ currents. Cannabinoid activation of CB_1_ has a similar inhibitory effect on vesicle release (Huang et al., 2007). GLU input to MCH neurons is conveyed by multiple receptors, including ionic (NMDAR and AMPAR) and mGlu1 and mGlu5. The activation of mGlu5 in MCH neurons leads to a potentiation of NMDAR responses through protein kinases A and C (PKA and PKC).

MCH-ir neurons are densely contacted by ORX neurons, with some single MCH-ir cells receiving contacts by up to 30 individual ORX-ir boutons. Both ORX_A_ and ORX_B_ have a postsynaptic excitatory effect over MCH cells, depolarizing their membrane potential and increasing spike frequency (**Figure 4A**). ORX_B_ has also a presynaptic effect, increasing GLUergic input to MCH cells (van den Pol et al., 2004) (**Figure 5**). The similar response amplitude of MCH neurons to ORX_A_ and ORX_B_ suggests that this signaling occurs through ORXR_2_, as ORXR_1_ responds poorly to ORX_B_ (Sakurai et al., 1998). Apergis-Schoute et al. (2015), however, provided evidence that the relationship between ORX and MCH may be more complex, since optogenetic stimulation of ORX neurons in LHA slices promoted inhibition of some MCH neurons through a presynaptic mechanism, increasing GABAergic input on MCH cells, while other MCH neurons were excited by ORX stimulus. Regardless, all these results suggest that these two LHA populations are connected, with ORX influencing close MCH neurons in multiple ways.

While some MCH neurons co-synthesize the CART (Elias et al., 2001) and nesfatin (Brailoiu et al., 2007; Fort et al., 2008), almost all ORX neurons co-synthesize Dyn or N/OFQ, both endogenous opioids (Maolood and Meister, 2010). The application of Dyn or N/OFQ hyperpolarizes MCH cells, increasing their input resistance (Li and van den Pol, 2006; Parsons and Hirasawa, 2011), probably through κ-opioid (κOR) and NOP receptors present in these cells (Parks et al., 2014) (**Figure 4B**). These results suggest that MCH neurons could potentially be stimulated by ORX and inhibited by Dyn and N/OFQ originated from the same fibers. Li and van den Pol (2006) and Parsons and Hirasawa (2011) report that MCH cells quickly desensitize to Dyn and N/OFQ, while the response to ORX remains largely unaltered after sequential applications. Through this mechanism, Dyn and N/OFQ could damp the MCH response to brief spontaneous activation of ORX cells, since only constant ORX input would surpass the desensitized inhibitory effect of Dyn and N/OFQ and activate MCH cells. In other words, these endogenous opioids provide the ORX-MCH circuit with a mechanism for noise filtering.

In addition to the chiefly LHA-centered MCH and ORX populations, neurons with distinct chemical signatures and broader distributions are also found in the LHA. One example is the TRH-ir neuronal population located in the LHA, that does not co-localize with MCH or ORX (Horjales-Araujo et al., 2014) and can also be identified in the PVH, VMH, DMH, suprachiasmatic, Pe and Arc hypothalamic nuclei (Brownstein et al., 1974). Besides its role in thyroid function regulation through the AH, TRH is also associated with the control of energy homeostasis through central pathways: icv injections decrease feeding and increase thermogenesis and locomotor activity (for a review on TRH functions, see Lechan and Fekete, 2006). The central effects of TRH are all antagonistic to MCH actions, suggesting that TRH-mediated inhibition of MCH neurons could account, at least partially, for the observed results. Indeed, Zhang and van den Pol (2012) report that TRH inhibit MCH neurons by increasing presynaptic GABAergic input to MCH neurons (**Figure 5**). In conclusion, TRH may modulate feeding behavior and energy expenditure by inhibiting MCH neurons in the LHA, and the source of this TRH input may be from the LHA itself or from other hypothalamic nuclei linked to energy balance, as the PVH, VMH, DMH, and Arc.

Recently, another population of LHA neurons has emerged an important mediator of energy homeostasis: Nts neurons that also contain LepR, known as Nts-LepR. These neurons are found in the LHA (Leinninger et al., 2011) and make synaptic contacts with local neurons (Louis et al., 2010; Leinninger et al., 2011). Although Leinninger et al. (2011) have shown that Nts-LepR neurons make inhibitory synaptic contacts with ORX neurons, but not MCH neurons, it is possible that a polysynaptic circuit may link Nts-LepR neurons to MCH. The selective ablation of LHA Nts-LepR neurons leads to a phenotype of increased body weight, but these mice have only a slightly increased food consumption when young. On the other hand, these animals have increased adiposity and decreased VO_2_ and ambulatory activity (Leinninger et al., 2011). This phenotype inversely mirrors that observed in *Pmch^-/-^* and *Mchr1^-/-^* mice, that show a reduction in chow consumption that is too small to explain their weight deficit, increased O_2_ consumption and increased ambulatory activity (Shimada et al., 1998; Marsh et al., 2002). These energy expenditure alterations seen in Nts-LepR mice are more compatible with overactivity of MCH neurons than exclusively of ORX. As Nts-LepR neurons are predominantly GABAergic (Leinninger et al., 2009; Goforth et al., 2014), synaptic spilling or GABA-mediated decrease of GLUergic input to MCH neurons may explain how these neurons modulate MCH neurons. As leptin serves as a metabolic signal for high energy reserves, the inhibition of MCH neurons by Nts-LepR neurons may be a mechanism to decrease MCH-mediated energy conservation in states of positive energy balance.

#### Arcuate Nucleus

The Arc is a classical component of the feeding behavior circuitry, as lesions in this nucleus and the adjoining VMH by gold thioglucose promote hyperphagia and obesity in mice (Debons et al., 1962). This nucleus contains neuronal populations that produce anorectic peptides, such as the POMC-derived α-MSH (Jacobowitz and O’Donohue, 1978; Elias et al., 1998; Ludwig et al., 1998) and CART (Koylu et al., 1997, 1998; Kristensen et al., 1998), as well as orexigenic peptides as the NPY (Allen et al., 1983; Clark et al., 1984) and the AgRP (Ollmann et al., 1997; Shutter et al., 1997). This nucleus is also influenced by insulin (van Houten et al., 1980; Niswender et al., 2003), glucose (Leloup et al., 1994; Muroya et al., 1999; Parton et al., 2007), and leptin (Mercer et al., 1996; Schwartz et al., 1996; Cheung et al., 1997), all of them capable of conveying the short- and long-term energetic state of the organism from the CSF to the Arc through the ME (Woods et al., 1979; Banks et al., 1996), positioning the Arc as the ideal receptor for these circulating metabolic markers.

Elias et al. (1998) showed that both major populations of the Arc, NPY/AgRP and POMC/CART, project densely to the LHA, with immunoreactive fibers in close apposition to MCH neurons. van den Pol et al. (2004) report that NPY inhibits MCH neurons, decreasing spike frequency and hyperpolarizing membrane potential, both postsynaptically through GIRK currents and presynaptically inhibiting GLU and GABA release to MCH neurons (**Figures 4B, 5**). To better understand these observations, it is important to consider that the LHA and the Arc present reciprocal connections. Direct injections of MCH in the Arc promote an increase in food consumption, placing this area as a site of MCH action (Abbott et al., 2003). Using hypothalamic cultures, these authors demonstrated that the addition of MCH to the culture induces a release of NPY, at the same time decreasing the amount of the anorectic α-MSH released by a hypothalamic explant. These data suggest that MCH projections from the LHA to the Arc may increase the release of the orexigenic peptide NPY and decrease the anorectic α-MSH, resulting in an overall increase in food consumption.

The results of Abbott et al. (2003) allow us to speculate that NPY inhibition of MCH neurons may be part of a negative feedback loop, with increased MCH activity on the Arc resulting in a depression of MCH neurons by NPY, avoiding excessive stimulation of this orexigenic circuit. Another possibility is that NPY–MCH interactions may be secondary to some other Arc-LHA circuits, as suggested by Segal-Lieberman et al. (2003) and van den Pol et al. (2004). Parallel to this negative feedback loop, a positive feedback loop may exist between MCH and the anorectic peptides of the Arc. Although the addition of α-MSH agonists or antagonists has no major electrophysiological effect on MCH neurons (van den Pol et al., 2004), the selective deletion of the *Pomc* gene results in a marked increase in *Pmch* mRNA, suggesting that POMC-derived peptides are capable of inhibiting *Pmch* expression (Challis et al., 2004). In this case, inhibition of α-MSH neurons by MCH would result in decreased inhibition of *Pmch* expression, potentially increasing the stimulation of NPY by MCH and reinforcing the negative feedback loop. It is important to consider, however, that these circuits may be polysynaptic, since there is little evidence for NPY and melanocortin receptors in MCH neurons (Liu et al., 2003; Parks et al., 2014). Therefore, these circuits may employ interneurons or even ORX neurons, since these are susceptible to multiple regulatory mechanisms by NPY (Fu et al., 2004). Finally, another possibility is that Arc projections interact with MCH-ir fibers at the PVH, a target nucleus that display important effector actions regarding feeding behavior and energy homeostasis.

#### Paraventricular Hypothalamic Nucleus

The PVH is one of the better-described nuclei in the whole hypothalamus. It has eight major subdivisions, with three magnocellular subdivisions containing neurons that primarily synthesize OXT and AVP, and five parvocellular subdivisions containing neurons that synthesize CRF and TRH, although we now know that many other neurochemical populations are present in the PVH with distinct distributions (Swanson and Sawchenko, 1983; Simmons and Swanson, 2008, 2009). In many ways, the PVH can be regarded as the motor nucleus of the endocrine system, directly releasing OXT and AVP in the NH and CRF and TRH in the portal circulation to modulate AH activity and influence two of the major neuroendocrine axis in the brain, the HPT and the HPA axis. Projections from these neurons, however, are not restricted to the hypophysis, as they project densely to other areas of the brain and all of them show, to some extent, modulatory effects.

To investigate if OXT and AVP neurons could exert effects through MCH neurons, Yao et al. (2012) evaluated the electrophysiological response of MCH cells to these neuromodulators. AVP directly excites MCH neurons through the V1a receptor, depolarizing their membrane potential and increasing spike frequency through NCX and the opening of TRPC (**Figure 4A**). AVP also acts presynaptically increasing GLU- and GABAergic input to MCH neurons (**Figure 5**). OXT provides excitatory input to MCH neurons as well, through the OXTR, depolarizing their membrane potential and increasing spike frequency through NCX (Yao et al., 2012) (**Figure 4A**). In addition to OXT and AVP, neuropeptides from the PVHp may also contribute to MCH-dependent modulation of ingestive behavior. As discussed in the previous section, TRH modulates MCH function and it is not possible, for now, to specify the exact source of this input to presynaptic GABA neurons in the LHA. Moreover, little has been described about the effects of CRF-synthesizing neurons over MCH cells albeit that, if such mechanism exists, it must involve polysynaptic circuits, since Parks et al. (2014) report that CRF receptors are not significantly found in MCH neurons.

#### Limbic and Mesolimbic

The Acb is an important component of the ventral *striatum*, participating in several processes, including reward and aversion (for a review, see Carlezon and Thomas, 2009). The Acb can be divided into two subdivisions, a core (AcbC) and a shell (AcbSh), with distinct connectivity, cellular composition and function. AcbSh activity has been associated with feeding inhibition, as electrical and optogenetic stimulation of AcbSh MSNs are capable of quickly interrupting a feeding bout; a subset of AcbSh neurons decrease their activity immediately before the onset of a sucrose solution consumption; and photoinhibition of dopamine-sensitive (D_1_) MSNs increases liquid fat intake (Krause et al., 2010; O’Connor et al., 2015).

AcbSh MSNs are GABA cells that project to the rostral parts of the LHA, suggesting the inhibition of orexinergic neurons of this area (MCH and ORX) as a putative mechanism for AcbSh-mediated inhibition of feeding. It is now understood, however, that AcbSh input to the LHA does not reach MCH or ORX neurons directly. Sano and Yokoi (2007) identified a group of GLU neurons in the anterior part of the LHA (LHAa) that receive dense projections from the AcbSh and project to the posterior part of the LHA (LHAp), suggesting that a GLU node exists between accumbal projections and neurons in the LHAp, where MCH neurons are located. O’Connor et al. (2015) identified a second pathway, reporting that D_1_-MSNs from the AcbSh project to the LHA and inhibit GABA interneurons in this area to promote accumbal inhibition of feeding. As an AcbSh|GABA-LHA|GABA-LHA|MCH circuit would be ultimately excitatory for MCH neurons, it is likely that additional nodes are present in this pathway. Further experiments will be necessary to elucidate the intra-LHA circuit that conveys accumbal information to MCH and ORX neurons.

The inhibition of MCH neurons by accumbal projections agrees well with the theory proposed by Kelley et al. (2005) that the AcbSh functions as a “sensory sentinel,” quickly inhibiting a feeding bout in response to an environment cue that may need the animal to respond to, such as a predator approaching, even if the animal’s metabolic state would drive him to continue feeding. Since MCH neurons project to the MCx (Elias et al., 2008) and decrease locomotor activity, an inhibition of these neurons could, at the same time, decrease the drive to feed and disinhibit the MCx, prompting the animal to show a motor response to the environmental cue.

Amygdaloid input to MCH neurons may also pay a role in the inputs to this system. Nakamura et al. (2009) report that anterograde tracer injections centered in the CeA reveal numerous varicose fibers in the LHA, chiefly on its dorsolateral most area. These fibers often make contact to MCH-ir and ORX-ir neurons and these terminals are immunoreactive for a GABA marker, suggesting an inhibitory property for these CeA projections.

#### Midbrain and Hindbrain

The midbrain contains some of the most important nuclei containing DA-synthesizing neurons in the whole CNS, including the A8, SN – A9, and VTA – A10 groups (Dahlström and Fuxe, 1963). These groups project densely to the forebrain, including the neocortex, the *striatum* and the diencephalon (Andén et al., 1966). Leibowitz and Brown (1980) showed that ventral A8 and A9 cells provide an important CA input to the perifornical area, and lesions of the A8 and A9 deplete the LHA of CA-ir, reduce the anorectic effect of the DA presynaptic drug amphetamine and potentiate the anorectic effect of DA itself. Alberto et al. (2011) and Conductier et al. (2011) report that DA produces hyperpolarization of MCH neurons through GIRK mediated outward currents, but DA receptors (D_1_ and D_2_) antagonists failed to induce similar currents, suggesting that the observed DA actions are not mediated by DA receptors (**Figure 4B**). Conductier et al. (2011) also found that DA can alter the presynaptic input to MCH neurons, with low concentrations of DA (1 μM) potentiating both GABA and GLU release through presynaptic D_1_ receptors, while high concentrations of DA (100 μM) inhibits GABA release through D_2_ receptors, with GABA effects more pronounced than GLU effects suggesting a predominantly inhibitory action for DA over MCH neurons (**Figure 5**). Both Alberto et al. (2011) and Conductier et al. (2011) found as well that the postsynaptic effect of DA depends on the adrenergic receptor α_2A_, since specific antagonists blocks DA-induced currents and α_2A_ is present in MCH neurons (Modirrousta et al., 2005).

These results are particularly noteworthy because the α_2A_ is also the receptor through which NE inhibits MCH action. The LHA is moderately innervated by NE terminals originated from the brainstem (Palkovits et al., 1974), with an important contribution from the lateral tegmental NE system (Moore and Bloom, 1979). The work of van den Pol et al. (2004) has provided evidence that NE promotes a hyperpolarization of the membrane potential and a decrease in spike frequency of active MCH neurons by mobilizing GIRK currents, and this effect is blocked by a selective α_2A_ antagonist (**Figure 4B**). NE also acts on the synaptic input to MCH neurons, decreasing GLUergic and increasing GABAergic input, resulting in an overall inhibitory action (**Figure 5**). Taken together, the results of van den Pol et al. (2004), Alberto et al. (2011), and Conductier et al. (2011) point to a synergistic convergence of both DA and NE systems through the α_2A_. This convergence may limit how much synergistic inhibition acts over MCH neurons, since DA inhibition would desensitize the receptors for NE inhibition and vice-versa. The DA and NE systems are, therefore, competitive elements instead of synergistic, even though both have inhibitory properties over MCH neurons.

Serotonin is a monoamine neurotransmitter synthesized in numerous groups of neurons of the brainstem and with widespread projections throughout the CNS (Dahlström and Fuxe, 1963; Steinbusch, 1981). Hypothalamic injections of 5-HT have been extensively correlated to a decrease in feeding (for a review, see Leibowitz and Alexander, 1998) through multiple receptors and different mechanisms (for a review, see De Vry and Schreiber, 2000). van den Pol et al. (2004) report that 5-HT hyperpolarizes MCH neurons and decreases spike frequency through postsynaptic mechanisms (**Figure 4B**). Taken into consideration, these data indicate that 5-HT may decrease feeding through the inhibition of MCH neurons in addition to 5-HT interactions with DA, although further experimental work is necessary to confirm this hypothesis and detect the specific 5-HT receptor subtypes responsible for this action.

The urocortins (UCN1, UCN2, and UCN3) are members of the mammalian CRF family, with a special affinity for the CRF receptor subtype 2 (Vaughan et al., 1995; Lewis et al., 2001; Reyes et al., 2001). UCN1 has been mapped in the albino rat by Kozicz et al. (1998) and Bittencourt et al. (1999), who have shown that the main sites of *Ucn1* mRNA expression and UCN1 synthesis are the EW and the superior olivary nucleus, both brainstem structures, with a broad distribution of UCN1-ir fibers throughout the CNS. Although the EW was initially recognized as a motor nucleus for vision related muscles, it is now recognized that the EW has two components: a preganglionic EW, responsible for this cholinergic motor control, and the centrally-projecting EW, involved in a series of functions such as feeding, stress and addiction (for a review in EW structure and function, we point the reader to Kozicz et al., 2011). Of special importance to us is the role of UCN1 in feeding behavior, as UCN1 acts as a powerful anorectic peptide in nanomolar concentrations (Spina et al., 1996) and diet/fasting can regulate *Ucn1* mRNA expression (Legendre et al., 2007; Xu et al., 2009). Júnior et al. (2015) have shown that UCN1-ir neurons or the EWcp send projections to the LHA and these fibers are in close proximity to MCH-ir neurons, so this UCN1|EWcp-MCH|LHA circuit could represent the pathway through which UCN1 influences feeding behavior. Although the low levels of CRF receptors in MCH could be taken as an evidence against this particular pathway, it is noteworthy that these receptors are found on the LHA (Chalmers et al., 1995), so the action of UCN1 could involve the modulation of GABAergic or GLUergic interneuronal input to MCH neurons. Further studies will be necessary to elucidate the physiological aspects of this circuit.

#### Circulating Factors

The MCH peptidergic system appears to be heavily influenced by leptin, a major circulating factor of satiety produced by adipocytes (Zhang et al., 1994; Campfield et al., 1995; Halaas et al., 1995; Pelleymounter et al., 1995). Qu et al. (1996) were the first to identify a relationship between MCH and leptin, reporting that *Lep^ob/ob^* mice have twice the level of *Pmch* mRNA than WT animals, suggesting a leptin inhibition of *Pmch* expression. Agreeing to that, obese rats with a point mutation in the long form of the *LepR* gene (Zucker rats *LepR^fa/fa^*) display higher levels of *Pmch* mRNA and decreased levels of *Mchr1* mRNA in comparison to lean rats, suggesting that deficient leptin signaling can upregulate the MCH system (Stricker-Krongrad et al., 2001). Likewise, Gavrila et al. (2005), studying humans, report that fasting (with a concomitant fall of circulating leptin levels) significantly increases serum levels of MCH, and supplementation with r-metHuLep prevents the raise of seric MCH. The mechanism through which MCH responds to leptin, however, must be polysynaptic, as studies using a reporter associated to the *Lepr* gene indicate that *Lepr-*expressing neurons are not MCH or ORX (Leinninger et al., 2009; Louis et al., 2010).

Other important factors for metabolism regulation are glucose and insulin. Although the periphery holds the machinery for glucose-mediated insulin production, and this machinery works efficiently to keep the blood levels of glucose in a strict range, we now include the CNS as an important sensor for these metabolic cues and as an effector to keep their physiological levels (Myers and Olson, 2012). The IR is widely distributed in the CNS (Mayer et al., 1978), with deleterious effects on energy homeostasis occurring after its specific deletion (Brüning et al., 2000). Hausen et al. (2016) provide evidence that MCH neurons can respond to insulin, reporting that 200 nM of it is capable of activating PIP3-dependent pathways and invoking action potentials in 6 of 13 neurons tested, while 3 neurons displayed a decrease in firing and the remaining neurons showed no alteration, suggesting that subpopulations of MCH neurons are responsive to insulin with different dynamics (**Figure 4A**).

In addition to insulin, Burdakov et al. (2005) provided evidence that glucose is also able to modulate MCH neurons. Although not as high as plasma levels, the brain is subjected to variations in glucose concentration that mirror those of the plasma, with a physiological range that goes from ∼0.7–2.5 mM and may reach as low as 0.2 mM in conditions of hypoglycemia and 5 mM in hyperglycemia (Routh, 2002). A subset of approximately 70% of MCH-ir neurons is excited by high levels of glucose (5 mM) and dose-dependently hyperpolarizes as glucose levels reach 0.2 mM (Burdakov et al., 2005; Kong et al., 2010). Kong et al. (2010) demonstrated that glucose sensing by MCH neurons works in a similar fashion to pancreatic β-cells, in which an increase in intracellular glucose (driven by an increase in extracellular glucose carried by the GLUT3 transporter) results in increased ATP production which, in turn, induces the closure of K_ATP_ with consequent depolarization, and this effect is dependent of SUR1-containing K_ATP_, establishing the SUR1 protein as a marker for glucose-sensitive MCH neurons (**Figure 4A**).

Although the activation of MCH in conditions of high glucose may seem counterintuitive, it is possible to fit it in a larger role for MCH in energy homeostasis. MCH is suggested to be involved in energy conservation, decreasing locomotor activity and metabolic parameters and increasing sleep in conditions of positive energy balance, thus its activation in response to high glucose (Burdakov et al., 2013). This led us to hypothesize that the orexigenic activity of MCH is not incompatible with that suggestion, when we consider the probable environment of an animal regarding food availability. It would be dangerous for animals to wait for their energy levels get low, as this could impair their capacity to obtain food, so it is beneficial for animals to eat periodically, even when their energy balance is positive. In most situations, animals have some awareness of their surroundings, and sources of food probably have been scouted, with animals preferentially occupying areas with abundant food availability. In that case, animals do not have to explore for food, so they may display decreased locomotor activity and longer periods of rest. Still, they must have some drive to feed periodically, and MCH could account for this effect, integrating not only glucose levels but also the other input discussed in this section. Food depletion of their environment or hazards (such as predators) may impair their ability to keep this constant state, in a situation that would lead to decreased levels of glucose. In this case, MCH would need to be shut off (low-glucose induced hyperpolarization) and a system that promotes food-seeking could be activated.

A possible component of this food circuit is ORX, as ORX-ir cells are excited by low levels of glucose (Burdakov et al., 2005) and their activation is also implicated in arousal (Alexandre et al., 2013), a necessary condition for active food-seeking. In this model, these two peptides could act in different aspects of feeding behavior: MCH is responsible for keeping a positive energy-balance state, while the ORX system “kicks in” when the animal reaches a negative energy balance and must return to a safer range. Since MCH is a “baseline stabilizer,” avoiding big fluctuations in energy homeostasis, MCH inactivation would not promote big alterations in feeding, since other ingestive-controlling systems would act nevertheless when the animal entered deficient energy states, possibly consuming an amount of food similar to that of an MCH-preserved animal. This appears to be the case, since the selective ablation of MCH-neurons in adult mice by diphtheria toxin promotes leanness and hyperactivity, but the animals display only a non-significant tendency to eat less than control mice (Whiddon and Palmiter, 2013).

Another possible peripheral signal that may influence MCH neurons is CORT, a glucocorticoid produced by the adrenal gland that freely crosses the BBB to influence the regulation of energy balance, with central and systemic administration of glucocorticoids promoting an increase in food intake and body weight (Freedman et al., 1986; Green et al., 1992; Tataranni et al., 1996; Zakrzewska et al., 1999). Drazen et al. (2004) have shown that, in ADX animals, the icv injection of MCH has a less potent effect than in sham animals regarding both food and fluid intake, and this effect is reversed in ADX animals that receive CORT supplementation in their water. These authors also report that hypothalamic expression of *Pmch* mRNA is decreased in ADX animals. This is in good agreement with the results of Viale et al. (1997) that showed the partial sequence of a glucocorticoid response element in the 5′-flanking region of the *Pmch* gene, which could account for the mechanism through which the absence of CORT after ADX promotes a decrease in *Pmch* mRNA expression.

Amylin is a pancreatic peptide hormone, co-synthesized with insulin by β-cells in response to feeding (Leffert et al., 1989), although central AMY has been described as a lactation-related peptide synthesized in the MPOA (Dobolyi, 2009). AMY has an anorectic action, suppressing feeding in both rats and mice, suggesting a post-prandial satiety effect and long-term lipostatic signaling (Lutz et al., 2001; Rushing, 2003). The intraperitoneal injection in rats of salmon calcitonin, an AMY receptor agonist, decreases the levels of LHA *Pmch* mRNA (Barth et al., 2003). This effect is probably not direct, since there is little evidence for AMY binding in the LHA (Sexton et al., 1994), suggesting that AMY signaling reaches MCH neurons through circuits that may include the AP (Lutz et al., 2001), VTA (Mietlicki-Baase et al., 2013, 2015) and LDTg (Reiner et al., 2017).

### Outputs

The regulation of feeding by MCH neurons is a complex mechanism that involves many aspects of this behavior, including the energy balance of the organism, metabolic rate, rewarding circuits, locomotor activity, arousal, spatial memory, olfactory cues and complex interactions with other peptidergic systems. In this section, we will highlight the main actions of MCH and its underlying connectivity, illustrated in **Figure 2B**.

#### Locomotor Activity

MCH-ir neurons project diffusely to numerous areas of the isocortex, including the SSCx, MCx, infralimbic, and Ins, as shown by Saper et al. (1986) using an antibody for α-MSH that was, as we now know, cross-reacting to MCH. This pattern of MCH projections was confirmed by Cvetkovic et al. (2003) using an antibody for salmonid MCH and further explored by Elias et al. (2008), that showed extensive innervation of the MCx by MCH neurons of the LHA. These authors also reported that a subset of these MCH-ir MCx-innervating neurons also innervate the PPTg, what may also contribute to a motor regulation through the mesencephalic locomotor region of this area. This circuit supports an action of MCH decreasing locomotor activity as reported in *Pmch^-/-^* and *Mchr1^-/-^* mice (Segal-Lieberman et al., 2003; Willie et al., 2008), as well as progressive (Alon and Friedman, 2006) and sudden (Whiddon and Palmiter, 2013) adult ablation of MCH neurons. Through this pathway, MCH neurons decrease locomotor activity and energy expenditure to revert states of negative energy balance or sustain states of positive energy balance.

#### Energy Expenditure

Locomotor activity is not the only way the CNS can modulate energy expenditure, since thermogenesis also play an important role in this aspect of homeostatic balance. The first link between MCH and energy expenditure control was drawn by Shimada et al. (1998), that noticed a reduction in body weight that surpassed the reduction in food intake in *Pmch^-/-^* animals. Although these animals did not have a lower rectal temperature when compared to WT mice, their rate of O_2_ consumption normalized for body weight was 20% higher. Mice lacking MCHR1 also display higher metabolic rates during the dark phase as measured by indirect calorimetry (Marsh et al., 2002).

Using a model of cold exposure, Pereira-da-Silva et al. (2003) showed that MCH is increased by almost 60% in cold-exposed animals. These animals display a series of physiological adaptations to the challenging temperature that include an increase in food consumption, fall of body weight and thermogenesis. These authors report that cold-exposed animals with impaired MCH synthesis have an increase in the molecular machinery necessary for BAT-dependent thermogenesis when compared to control animals, suggesting that MCH inhibits BAT thermogenic activity, potentially conserving energy for homeostatic purposes. To investigate the chemical signature of the hypothalamic circuits modulating BAT activity, Oldfield et al. (2002) injected a polysynaptic viral tracer in the BAT of adult rats combined to immunohistochemistry for different neuropeptides synthesized in the hypothalamus. Third-order MCH-ir neurons were found in the LHA, suggesting that MCH neurons provide input to the BAT through brainstem nuclei.

These results are reinforced by the works of Guesdon et al. (2009) and Chung et al. (2010). Guesdon et al. (2009) report that intraventricular injections of MCH decrease lipid oxidation and Chung et al. (2010) demonstrated that *Mchr1^-/-^* animals are resistant to weight gain when exposed to HFD due increased lipid metabolism. Furthermore, Verty et al. (2010), using temperature-sensitive telemetry probes, registered a significant increase in BAT temperature after intraventricular injection of an MCHR1 antagonist. All these data suggest that LHA MCH neurons polysynaptically decrease thermogenesis in BAT to reduce lipid metabolism and energy expenditure, an important mechanism to sustain a positive energy balance or revert a scenario of negative energy balance.

#### Autonomic Modulation

Saper et al. (1976) have shown that LHA neurons project both to autonomic centers of the brainstem, such as the dorsal motor nucleus of the vagus and the NTS, and directly to the SpCd, including the ImC in thoracic levels, suggesting a descending hypothalamo-autonomic pathway that access directly the preganglionic cells of the parasympathetic and sympathetic systems. Since MCH-ir fibers from the LHA also sparsely innervate the autonomic nuclei of the brainstem and all levels of the SpCd (Kohler et al., 1984; Bittencourt et al., 1992; Bittencourt and Elias, 1998), this represents a possible pathway through which MCH may integrate ingestive behavior to autonomic function, although further evidence is necessary to establish if there is a direct contact between ganglionic cells and MCH neurons, possibly through specific viral tracers. More recently, Pérez et al. (2011) injected a polysynaptic viral tracer in the SAL and MAS of mice and discovered that MCH-ir neurons of the LHA provide inputs to brainstem nuclei involved in the control of these structures (MoV – MAS innervation; 7N and SuS – SAL innervation). Through this innervation, LHA MCH-containing neurons can gate two important preparatory responses to feeding: salivation, an autonomic response necessary for bolus formation and swallowing, and mastication, a motor response which involves complex coordination between muscles of the jaw, face, and tongue. Another interesting aspect of this MCH input is the fact that both salivation and mastication are under the control of higher-order cortical centers (Thexton, 1992; Hubschle et al., 1998), and MCH could potentially integrate this higher-center input and provide the output to lower brainstem nuclei.

An indirect mechanism for this feeding-autonomic coupling is through the PVH. An important number of labeled cells are found in the PVH after retrograde tracer injection in the SpCd, suggesting that this nucleus also acts over autonomic systems (Hancock, 1976; Saper et al., 1976; Ono et al., 1978; Blessing and Chalmers, 1979; Hosoya and Matsushita, 1979). The PVH neurons that project to the SpCd and to the dorsal vagal complex are concentrated in the dorsal, lateral, and medial parts of the PVHp (Swanson and Kuypers, 1980), regions that receive more MCH-ir innervation than the PVHm (Bittencourt et al., 1992). Since injections of MCH restricted to the PVH without spilling to the ventricular system promote an increase in feeding behavior (Abbott et al., 2003), this could represent another node through which MCH integrates feeding behavior and autonomic function, triggering the necessary autonomic preparatory processes to feeding.

#### Thyroid Modulation

Kennedy et al. (2001) suggest that MCH could be part of the peptidergic modulation of the HPT axis. After icv injections of MCH, there is an *in vivo* decrease in the plasmatic levels of TSH (10 and 60 min after injection). *In vitro*, the addition of MCH and NEI in hypothalamic cultures promotes a decrease in the levels of TRH, and MCH co-applied with TRH decreases the secretion of TSH in cultures of hypophysaire cells when compared to TRH alone. MCH may act both through a direct and an indirect pathway to control the HPT axis activity. Directly, MCH-ir fibers from the LHA could innervate TRH-ir neurons in the PVH, decreasing their secretory activity. In the indirect pathway, MCH-ir projections to the Arc could potentially act over the α-MSH/CART and NPY/AgRP systems to promote downstream alterations in TRH synthesis. There is anatomical support for both pathways. Both MCH-ir fibers and MCHR1 immunoreactivity are found in the PVH of rats (Bittencourt et al., 1992; Hervieu et al., 2000; Saito et al., 2001) and a reporter to associated to *Mchr1* can be detected in TRH-ir cells of the PVH (Chee et al., 2013). An Arc mediated pathway is supported by the observation that the neonatal ablation of the Arc abolishes the decrease in PVH| TRH resulting from fasting (Legradi et al., 1998). NPY-ir and AgRP-ir nerve terminals contact TRH-ir neurons in the PVH, and the main source for this innervation is the Arc (Legradi and Lechan, 1998, 1999). A similar innervation of pro-TRH mRNA expressing-neurons is also observed for α-MSH and CART fibers (Fekete et al., 2000). Regardless of the pathway, considering the energy conservation effects of MCH, it is likely that this peptide acts on the HPT axis to decrease energy expenditure.

#### Circulation

There are few studies that examined the levels of MCH in the bloodstream. Evaluating the possibility that MCH was produced and released by adipocytes, Bradley et al. (2000) did not detect *Pmch* mRNA in these cells, but they detected MCH in the plasma through a RIA. Stricker-Krongrad et al. (2001) evaluated the amount of MCH in the serum of Zucker rats and found that MCH levels were increased in the obese animals compared to the lean ones. On the other hand, in Wistar rats with obesity resulting from electrolytic VMH or PVH lesions, there was a decrease in the intensity of MCH staining in the LHA (9.3% for VMH lesions and 21.9% for PVH lesions), but the decrease in serum MCH levels did not reach statistical significance (Sun et al., 2004). It is important to consider, however, that Sun et al. (2004) used densitometry in LHA slices after immunohistochemistry to quantify the alterations in MCH immunofluorescence, a method that allows the detection of general differences but renders impossible to define if subsets of neurons had alterations. Therefore, it is still possible that some neurons displayed a decrease in MCH synthesis in response to the lesions, but the neurosecretory neurons kept stable levels of synthesis.

Gavrila et al. (2005) examined serum levels of MCH in humans, evaluating correlation factors for diverse parameters such body fat, leptin, estrogen and testosterone, dietary patterns and fasting. These authors found a direct correlation between serum MCH and fat mass and fat percentile, but no correlation could be drawn between MCH levels and estrogen, testosterone or dietary parameters. Working also with humans, Carnier et al. (2010) evaluated obese adolescents with eating disorders after 6 months and 1 year of interdisciplinary therapy, and observed a short-term increase of MCH in the blood of these patients followed by a decrease after 1 year of therapy. These authors suggest that the short-term increase in energy demand upregulate the release of MCH and the long-term correction of dietary habits promote a lower MCH baseline. During the preparation of this review, a new study about circulating MCH in humans was published by Naufahu et al. (2017). In this study, a reliable RIA was developed and tested in over 200 humans of both sexes and different body fat profiles, revealing a plasma MCH baseline of 19.5–55.4 pg/ml and some differential regulation of MCH that depends on both gender and adiposity, although further studies are necessary to fully elucidate how that regulation occurs.

The data regarding circulating MCH must be examined with caution. One major caveat is that Waters and Krause (2005), in a letter to the editor addressing the work of Gavrila et al. (2005), report that the commercial RIA used in most of these studies for MCH has non-specific reactivity with serum elements, since samples from *Pmch^-/-^* mice resulted in a positive signal. However, it is now hard to dispute that MCH is found in physiological levels in the bloodstream after the experiments ran by Naufahu et al. (2017). Another important aspect about circulating MCH is that its source is still unknown. Several peripheral tissues synthesize MCH (Hervieu and Nahon, 1995), and the role of this synthesis is, in many cases, still not associated with specific functions. Viale et al. (1997) showed that peripheral MCH has a different structure when compared to central MCH, as it is processed differently and its sequence combines central MCH and NEI. Since many peripheral tissues display MCHR1 mRNA (Saito et al., 1999; Hill et al., 2001), it is highly likely that MCH released in the bloodstream from one or more sources can reach those tissues and play modulatory roles. The study of MCH as a secreted factor in the bloodstream represents, therefore, a prolific field of MCH study.

#### Reward

In an oversimplification, feeding behavior is stimulated by two main drives: a metabolic drive, guided by homeostatic cues aiming to keep the energy balance of the animal, and a reward drive that involves complex emotional aspects and is directly linked to the subjective experience of pleasure. The reward aspect of feeding is a major component of that behavior, playing a preeminent role in our modern society. Rats are willing to expose themselves to aversive stimuli, such as foot-shock, painful heat and intense cold to obtain palatable food, such as candies and soda (Cabanac and Johnson, 1983; Foo and Mason, 2005; Oswald et al., 2011). As the complex relationship between metabolic and reward aspects has been thoroughly examined by others (Saper et al., 2002; Berthoud, 2011; Ferrario et al., 2016), in this review, we will focus on the role that MCH may play coordinating these different drivers.

The motivation and reward system involves a complex circuit with many relevant nodes, including the prefrontal cortex, *striatum*/Acb and the VTA. One of the regions with highest *Mchr1* mRNA expression is the Acb (Hervieu et al., 2000; Saito et al., 2001) and MCH fibers are found in this area (Bittencourt et al., 1992), especially numerous in the septal pole of the AcbSh (corresponding to the dorsocaudomedial AcbSh) (Haemmerle et al., 2015). Haemmerle et al. (2015) also report that accumbal MCH-innervation originates from neurons in all major hypothalamic areas containing MCH-ir neurons, with the highest rate of neurons simultaneously labeled for MCH and retrograde tracer found in the IHy. These results are in good agreement to those of Kampe et al. (2009) that report AcbSh-projecting MCH neurons in the lateral hypothalamus that also project to the CgCx/Ins.

MCH influences feeding behavior through the AcbSh, as MCH injected in this area promotes an increase in chow consumption, while an MCHR1 antagonist injected in this area decreases feeding (Georgescu et al., 2005). These authors also report that MCHR1 co-localizes to enkephalin and Dyn in the AcbSh, suggesting that MCH may influence the opioid system through these cells, a possible crossroad between MCH and the reward system (Holtzman, 1974; Frenk and Rogers, 1979). These results are supported by the work of Mul et al. (2011) that, using the only published model of *Pmch^-/-^* rat, found that the MCH signal on the AcbSh is important to keep the motivational aspects of the feeding behavior. Therefore, this hodological aspect between the LHA, the AcbSh and limbic areas is fundamental for the characteristic control of energy balance and to increase motivational or incentive-related aspects of food consumption.

A new facet of MCH actions on reward was provided by Karlsson et al. (2012). Using an MCHR1 antagonist combined with self-administration of sucrose (caloric and sweet) or saccharin (just sweet), these authors found that antagonist administration reduces sucrose self-administration, but not saccharin-reinforced lever-pressing, indicating that MCH conveys rewarding signals from the caloric content of consumed food. The antagonist administration also decreased cue-induced reinstatement of sucrose seeking, confirming that MCH participates in the rewarding properties of sucrose.

Domingos et al. (2013) extended these result employing optogenetic manipulation of MCH neurons in mice. Photostimulation of MCH neurons is able to revert the innate preference of mice for sucrose over saccharin and stimulate DA release in the *striatum*. A similar effect is observed in sweet-blind mice, suggesting that taste is not necessary for MCH-dependent preference for caloric ingestion. Contributing to these effects may be an activation of DA neurons in the VTA, as MCH ablation reduced FOS synthesis in this area. The VTA is densely innervated by MCH (Bittencourt et al., 1992) and MCHR1 is found in low (Saito et al., 2001; Pissios et al., 2008) to moderate (Hervieu et al., 2000) numbers in this area. CART/MCH terminals are found in the VTA, and CART-ir fibers contact DA neurons, suggesting that MCH and CART may work in tandem to modulate DA release by VTA neurons (Dallvechia-Adams et al., 2002), although electrophysiological data suggests otherwise (Korotkova et al., 2003). One possible explanation is that CART and MCH may not act directly on DA neurons, but modulate their auto inhibition. CART impairs DA-D_2_ binding when co-applied with stimulatory substances (Jaworski et al., 2003; Kim et al., 2003; Moffett et al., 2011), and DA neurons are auto inhibited through D_2_ (Johnson and North, 1992; Momiyama et al., 1993), suggesting that CART may decrease DA-dependent inhibition of DA neurons, increasing their activity. Since MCH can depress the presynaptic machinery of release (Gao and van den Pol, 2001), MCH released with CART in the synapse could depress further CART release, preventing too much disinhibition of DA neurons. A similar mechanism to this has an experimental basis in the work of Yang and Shieh (2005), on which MCH impaired CART stimulation of DA activity in the Acb. Further experimental evidence, however, is necessary to test if this is the mechanism of action for MCH, CART, and DA in the VTA.

The interactions between MCH and DA are not limited to the VTA, however, as these two may also interact directly on the Acb. When Chung et al. (2009) applied MCH or D_1_ and D_2_ agonists separately in the AcbSh, there was no change in animal behavior, but when the three were co-applied there was a potentiation of the cocaine rewarding response. Furthermore, *Mchr1^-/-^* animals displayed resistance to the rewarding effects of cocaine, in a similar way to the pharmacological blockade of this receptor. More work will be necessary to fully elucidate the interaction between MCH and DA, however, as Smith et al. (2005) observed upregulation of D_1_ and D_2_ in the AcbSh of *Mchr1^-/-^* mice and hyper sensibility to amphetamine, while methamphetamine responses were attenuated after injections in the AcbSh of MCH and amplified after MCHR1 agonists injections (Sun et al., 2013). Albeit Chung et al. (2009) suggest that these apparent contradictions can be explained by the different mechanisms of cocaine and amphetamine action, more evidence is necessary to confirm this and to allow us to understand the role that MCH may play in reward and addiction.

As a final consideration about MCH and reward, although alcohol consumption is not the theme of this review, both drug and food consumption have a common ground that is the reward motivation. Alcohol is a particularly interesting model, as it is both a stimulatory factor of the reward system and a source of caloric nutrient. Duncan et al. (2005) found an increase in 10% alcohol and isocaloric sucrose solutions intake after 3V MCH injections, suggesting that MCH may drive the pursuit of these substances for their rewarding or caloric value. Morganstern et al. (2010a) developed on these results, demonstrating that injections of MCH restricted to the PVH and Acb were able to increase alcohol consumption, suggesting those nuclei as downstream targets of MCH action on alcohol consumption. The exact mechanism by which this MCH modulation happens was, however, contentious, as some reports suggested that MCHR1 was not involved in the alcohol consumption-promoting effect of MCH (Duncan et al., 2006, 2007), while others found a potent decrease in alcohol consumption and reinstatement after an MCHR1 antagonist treatment (Cippitelli et al., 2010). Recently, Karlsson et al. (2016) reported that MCH and MCHR1 have a dual role in the regulation of alcohol intake through mechanisms related both to caloric intake and reward motivation. Further experiments on MCH alterations linked to alcohol consumption, however, are still necessary to provide an overarching explanation for all results reported in the literature. Understanding the mechanisms through which MCH modulates alcohol consumption may provide insightful views on addictive and excessive behaviors.

#### Foraging/Predation

The participation of MCH in the pre-ingestive steps of feeding is perhaps its most elusive aspect so far. Albeit the *ad libitum* diet used in most experimental designs is convenient to standardize parameters, it also creates a highly artificial setup for the animal. In its natural environment, the animal must constantly make decisions regarding food acquisition while factoring elements such as its current energetic availability compared to other needs, foreseen energetic necessity, proximity to food sources and the risk of being eaten by a predator or injured by another environmental hazard. Some animals must also decide when to hunt for prey, since predation may reward it with metabolic needs, if successful, but may also incur in wasted energy if a failure.

Among the many complex elements of foraging and predation, there are three aspects to which MCH may be associated to: sensory integration, decision-making, and memory. The first role proposed for MCH in the mammalian brain was over the modulation of auditory stimuli, by Miller et al. (1993). Using an auditory gating paradigm, these authors have shown that the suppression of a redundant auditory signal is abolished after MCH addition to the CSF, suggesting that MCH can induce a state of higher vigilance at the cost of decreased focus on a single signal. Besides auditory stimuli, Adams et al. (2011) provided evidence that MCH participates in the integration of olfactory stimuli through numerous MCH-ir fibers that are found in olfactory-linked structures such as the Pir and the OB. Using *Pmch^-/-^* mice, these researchers observed a decreased capacity for them to find food using olfactory cues, suggesting that MCH may display an important role in the search for food. MCH is, therefore, an important actor in the gating of sensorial information, altering perceptual properties of the animal (such as vigilance and focus) depending on the available energy stores.

Regarding decision-making, there is a paucity of functional information relating it to MCH. Projections arising from MCH neurons in the IHy and tuberal LHA densely and diffusely reach the PAG (Elias and Bittencourt, 1997). Comoli et al. (2003) have shown that the lateral aspects of the PAG are involved in predatory behavior, as FOS synthesis in this area is increased in rats after cockroach predation compared to control animals. It is possible, therefore, that MCH-ir projections to the PAG may influence predatory behavior, but experimental evidence is necessary to establish the PAG-mediated actions of MCH.

The third important aspect of foraging is memory, as animals that can adequately remember the location of food sources will have a survival advantage. The HF has been thoroughly implicated in memory (Scoville and Milner, 1957; Zola-Morgan et al., 1986; Squire, 1992) and is one of the major targets of MCH-ir fibers and MCHR1 synthesis in the rat brain (Bittencourt et al., 1992; Hervieu et al., 2000; Saito et al., 2001). Lima et al. (2013) provided a more detailed description of the MCH input to the HF, describing a higher density of MCH-ir fibers in the dorsal HF, especially in the CA3 field, and the presence of MCH-ir fibers plexuses around GABAergic basket cells in CA1 and CA3, in addition to apparent contacts between MCH-ir fibers and HF-projecting cholinergic cells of the MS.

Several authors have provided evidence that MCH plays a role in HF modulation. After CA1 MCH injections, rats show: improved acquisition and consolidation in an inhibitory avoidance test (Monzon et al., 1999); a decrease in the levels of nitric oxide and its second messenger cGMP (Varas et al., 2002a); and a facilitation in long-term potentiation through alterations in the NMDA receptor-gated channel (Varas et al., 2002b, 2003). Adamantidis et al. (2005) provided further evidence for these memory-facilitation actions of MCH by reporting that *Mchr1*^-/-^ mice have worse memory retention of aversive stimuli and decreased response of pyramidal cells to NMDA in the CA1. Pachoud et al. (2010) expanded the roles of MCH in HF plasticity, reporting that *Mchr1^-/-^* mice have decreased output from excitatory GLUergic Schaffer Collaterals from the CA3 to the CA1 through both AMPA- and NMDA-mediated responses. In summary, these results point to MCH influencing GLUergic transmission in the CA1 field through both presynaptic mechanisms and the increase of NMDAR and AMPAR.

While most studies focused on the effects of MCH in the CA1 field, Sita et al. (2016) investigated the effects of MCH immunoneutralization centered on the CA3. Animals with impaired MCH signaling in the CA3 were more effective recalling the place where food was buried during the training phase, while control animals took longer and dug in other sites before attempting the right quadrant. The most reasonable explanation for the observed results is that animals could perceive olfactory cues indicating that there was no food in the test trial, so control animals searched for food in the arena while MCH-impaired animals were unable to incorporate these cues to the previously stored information.

What circuit could underlie the observations of Sita et al. (2016)? According to Easton et al. (2012), there is a dichotomy between the encoding of new afferent information into preexisting memories, a phenomenon dependent of the entorhinal cortex and the CA1 field, and the retrieval of memories, based on CA3–CA1 interactions. Therefore, the inhibition of MCH in the CA3 may have favored this latter circuit, allowing the animal to better recruit the location of the buried empty petri dish, but impairing its ability to perceive the absence of food, indicating that normal MCH activity in the CA3 is necessary for regular integration of sensory information to acquired memories, in a similar fashion to cholinergic input (Easton et al., 2012).

## Other Behaviors

**FIGURE 6:**
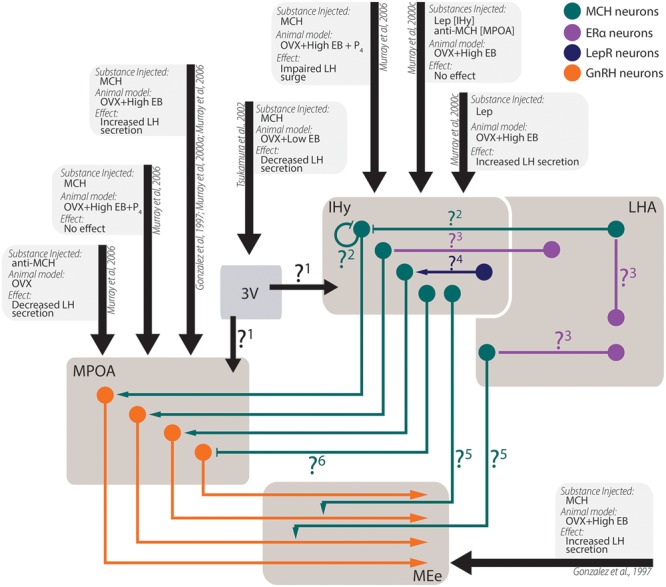
**Experimental data from *in vivo* experiments regarding MCH actions on sexual physiology.** Simplified schematic representation of the main findings regarding MCH actions after intracerebroventricular injections in areas related to sexual physiology and the circuit underlying these observations. Several questions remain open regarding the pathways necessary for the observed results: **1**. After intraventricular injections, a decrease in LH secretion was observed, although several other authors report increases in LH secretion after intranuclear injections. It is unknown, at this time, if this contradictory observation stems from MCH action on areas that may negatively modulate LH secretion or if the low EB milieu employed by the authors may have contributed to the observed differences; **2**. MCH injections centered on the IHy impair the LH surge expected after OVX+EB+P_4_ treatment, suggesting that auto-receptors or extra-IHy MCH inputs may negatively modulate LH release; **3**. Although physiological E_2_ levels are necessary for MCH-dependent modulation of LH secretion, MCH neurons do not co-localize with ERα, suggesting that E_2_ action over MCH neurons depend on polysynaptic circuits. These circuits are yet to be demonstrated; **4**. Leptin injected in the IHy increases MCH dependent-LH secretion, but MCH neurons do not co-localize to LepR, suggesting that polysynaptic circuits also convey leptinergic information to MCH neurons; **5**. MCH-ir axons are found near GnRH fibers, and the density of MCH fibers in the MEe vary according to the estrous cycle of female, but the source of these MCH-ir fibers is still unknown; **6**. Although several groups reported that MCH increases LH secretion by GnRH cells, electrophysiological data indicated that MCH hyperpolarizes GnRH neurons through postsynaptic mechanisms, therefore the exact mechanism for MCH-dependent LH secretion is uncertain.

The actions of MCH on behaviors not related to energy homeostasis are less understood, although recent developments began to shine some light in these other actions (**Figure 6**). There is ample morphological support for MCH actions over sexual behavior, as MCH-ir fibers are found in multiple relevant areas such as the AVPV, the MPOA, the AHA, and the ME (Bittencourt et al., 1992), where they may contact GnRH-ir neurons (Williamson-Hughes et al., 2005; Ward et al., 2009; Wu et al., 2009; Skrapits et al., 2015) and fibers (Williamson-Hughes et al., 2005; Ward et al., 2009). This MCH–GnRH interaction may underlie the modulation of MCH over LH release, although the exact mechanism through which this modulation occurs is still contentious (Gonzalez et al., 1997; Murray et al., 2000a, 2006; Tsukamura et al., 2000; Wu et al., 2009). In this regard, the MPOA and the IHy seem to play an important role (Murray et al., 2000b,c, 2006).

Not only MCH appears to impact the sexual physiology, but steroidal hormones seem to influence the MCH peptidergic system as well. In OVX rats supplemented with EB, the orexigenic effects of MCH are impaired in comparison to male and OVX-only rats, an observation that can be extended to physiological conditions (Messina et al., 2006; Santollo and Eckel, 2008, 2013). These effects of E2, however, appear to be dependent on polysynaptic mechanisms, since MCH neurons are close to ERα-containing neurons, but they do not co-localize (Muschamp and Hull, 2007; Santollo and Eckel, 2013). Steroidal cues also play a role in MCH fiber density, as the number of MCH-ir fibers in the external layer of the ME (MEe) is increased in the diestrus and proestrus, in sharp contrast to what is observed during estrus or in males (Chiocchio et al., 2001; Gallardo et al., 2004).

In conclusion, although there is an important amount of information regarding the relationship between MCH and sexual behavior, especially through the modulation of gonadotropin release, further experiments will be necessary to understand the exact mechanism of MCH action in animals in their natural environment, in light of the complex influence that steroidal hormones and sites of action appear to exert. It is noteworthy, as others have pointed (Naufahu et al., 2013), that MCH actions on sexual behavior probably incur in important survival gains by better coordinating the time for animals to engage in reproductive behaviors, since doing so in states of negative energy balance could incur in danger to the animal’s life and their potential offspring.

There is also evidence for MCH actions on maternal behavior and the lactation period. The very first functional clue was presented by Parkes and Vale (1993), that applied MCH and NEI in a NH cell culture and found that the amount of OXT secreted increases around 188 and 245%, respectively. Adams et al. (2011) described that female *Pmch^-/-^* mice show a higher litter postpartum mortality (31.1 ± 13.6% *Pmch^-/-^* vs. 7.3 ± 4.2% WT), an observation that they attribute to the reduced capability of the female to integrate olfactory stimuli. Alvisi et al. (2016) report that pup-suckling stimulus does not elicit MCH neurons activation or increase the number of MCH-ir neurons in the MPOA, suggesting that hormonal and other sensorial cues have prevalent interactions modulating the MCH system during this period. Recently, Benedetto et al. (2014) and Alachkar et al. (2016) demonstrated that MCH has an important role in the expression of maternal behavior through the MPOA, acting as a promoter of maternal behavior in the early postpartum period and a selective inhibitor of appetitive components of this behavior at late stages. It is noteworthy that Alachkar et al. (2016) describe impaired maternal aggression in *Mchr1^-/-^*, while Adams et al. (2011) report that *Pmch^-/-^* animals have increased levels of aggression when on a group and faster initial aggressive response. Besides those two works, very little is known about a possible role for MCH in aggression and defensive behavior, so further experimental investigation is necessary to better elucidate this aspect of the MCH peptidergic system.

Although not motivated behaviors *per se*, sleep and arousal are essential processes with an important interplay with motivated behaviors, especially through hypothalamic circuits (Saper et al., 2001, 2005). Intraventricular injections of MCH increase the amount of time spent by rats in REM and slow-wave sleep (Verret et al., 2003). Likewise, the subcutaneous treatment with an MCHR1 antagonist decreases the amount of REM sleep (Ahnaou et al., 2008). Recently, Vetrivelan et al. (2016) demonstrated that MCH neuronal activity increases REM sleep, but MCH neurons are necessary for the normal wake-REM sleep rhythm. MCH actions on sleep depend on projections to the DR (Lagos et al., 2009, 2011), oral pontine reticular nucleus (Torterolo et al., 2009), horizontal limb of the diagonal band of Broca (Lagos et al., 2012), ventrolateral preoptic area (Benedetto et al., 2013) and to the *locus coeruleus* (Monti et al., 2015). Sleep-promoting actions of MCH may be important to reduce activity and conserve energy in states of negative energy balance, when the animal has a propensity to increase arousal to maximize foraging. The results of Willie et al. (2008) suggest that this is the case, as fasted *Pmch^-/-^* animals have increased activity and exaggerated REM sleep time reduction.

## Concluding Remarks

In this review, we highlighted the circuitry that underlies the integrative functions of MCH. The MCH circuitry is an extremely relevant tool because it allows us to dissect some of the multiple inputs that reach the hypothalamus and the many targets that can be affected by this system. However, this exactly plethora of connections makes this peptidergic system overwhelmingly hard to study and to comprehend, as each experimental manipulation affects multiple physiological variables. It is not uncommon, therefore, to observe slightly distinct experimental approaches resulting in widely different results, sometimes to the point of an apparent contradiction between obtained data. Although diverse reasons can be pointed to explain this, it is remarkable to us that studying the MCH neuron as a single entity may be hampering the field. Studies like those of Burdakov et al. (2005) and Hausen et al. (2016) tell us that subsets of MCH neurons differ in respect to their electrophysiological responses to varied stimuli, while studies like those of Elias et al. (2008) and Kampe et al. (2009) provide evidence for differential projection fields among MCH neurons. This may be in the heart of our inability to turn MCH into a valid pharmacological target to treat obesity or psychiatric disorders (Méndez-Andino and Wos, 2007; Högberg et al., 2012), as the desirable effects of MCHR1 antagonism are overshadowed by unwanted side effects. Not everything is grim in the future of MCH study, however, as works that better characterize the morphology, the hodology and the chemical signatures of the LHA, such as those of Swanson et al. (2005), Hahn (2010), and Hahn and Swanson (2015), are important steps in the better characterization of the intrinsic properties of subsets of MCH neurons. Once we understand their variability, we may be able to design drugs to act only on the desired aspects of the MCH peptidergic system and preserve those that must not be manipulated. Accurate morphological data, nevertheless, continues to be an essential tool for the better understanding of peptidergic systems and of the integrative properties of the hypothalamus as a whole.

## Author Contributions

GD and JB contributed with the writing of this article and approved it for publication.

## Conflict of Interest Statement

The authors declare that the research was conducted in the absence of any commercial or financial relationships that could be construed as a potential conflict of interest. The reviewer SEK and handling Editor declared their shared affiliation, and the handling Editor states that the process nevertheless met the standards of a fair and objective review.

## Abbreviations

3V: third ventricle
5-HT: serotonin
5-HT_?_: unspecified serotonin receptor
7N: nucleus of the cranial nerve VII
A10: ventral tegmental area dopaminergic group
A13: incerto-hypothalamic dopaminergic group
A8: retrorubral dopaminergic group
A9: *substantia nigra* dopaminergic group
ac: anterior commissure
Acb: *nucleus accumbens*
AcbC: *nucleus accumbens*, core subdivision
AcbSh: *nucleus accumbens*, shell subdivision
ACh: acetylcholine
ADX: adrenalectomized
AgRP: agouti-related protein
AH: adenohypophysis/anterior lobe of the pituitary
AHA: anterior hypothalamic area
AMPAR: AMPA ionotropic glutamate receptor
AMY: amylin
Amyg: amygdaloid nuclei
AON: anterior olfactory nucleus
AP: *area postrema*
Arc: arcuate nucleus
AV: anteroventral thalamic nucleus
AVP: vasopressin
AVPV: anteroventral periventricular nucleus
B7: dorsal raphe serotonergic group
B8: median raphe serotonergic group
BAT: brown adipose tissue
BNST: bed nucleus of the *stria terminalis*
CA: catecholamine
CA1: CA1 field of the hippocampus
CA2: CA2 field of the hippocampus
CA3: CA3 field of the hippocampus
CART: cocaine- and amphetamine-regulated transcript
CB_1_: cannabinoid receptor type 1
cc: *corpus callosum*
CeA: central nucleus of the amygdala
CG: central gray of the spinal cord
CgCx: cingulate cortex
CORT: cortisol/corticosterone
cPPTg: caudal pedunculopontine tegmental nucleus
CPu: caudate putamen
CRF: corticotrophin-releasing factor
D_1_: dopamine receptor subtype 1
D_2_: dopamine receptor subtype 2
DA: dopamine
DG: dentate gyrus
DMH: dorsomedial hypothalamic nucleus
DR: dorsal raphe nucleus
DTM: dorsal tuberomammillary nucleus
Dyn: dynorphins
EB: estradiol benzoate
ERα: estrogen receptor type α
EW: Edinger–Westphal nucleus
EWcp: centrally projecting Edinger–Westphal nucleus
f: fornix
FOS: FOS proto-oncogene, AP-1 transcription factor subunit
GABA_A_: GABA ionotropic receptor
GIRK: G protein-coupled inwardly rectifying K^+^
GLUT3: glucose transporter 3
GnRH: gonadotropin-releasing hormone
GP: *globus pallidus*
Hab: habenular nuclei
*Hcrt*: Orexin/hypocretin gene
HF: hippocampal formation
HPA: hypothalamus-pituitary-adrenal
HPT: hypothalamus-pituitary-thyroid
Hyp: hypothalamic nuclei
IC: inferior colliculus
IHy: incerto-hypothalamic area
ImC: intermediolateral column of the spinal cord
Ins: insular cortex
IR: insulin receptor
K_ATP_: ATP-sensitive potassium channel
LD: laterodorsal thalamic nucleus
LDTg: laterodorsal tegmental nucleus
*Lep*: leptin gene
LepR: leptin receptor
*LepR*: leptin receptor gene
LG: lateral geniculate nucleus
LH: luteinizing hormone
LHA: lateral hypothalamic area
LHAa: anterior part of the LHA
LHAp: posterior part of the LHA
LM: lateral mammillary nucleus
LPOA: lateral preoptic area
LS: lateral septal nucleus
mACh: metabotropic ACh receptor
MAS: masseter muscle
MCH: melanin-concentrating hormone
MCH1: teleost melanin-concentrating hormone subtype 1
MCH2: teleost melanin-concentrating hormone subtype 2
MCHR1: MCH receptor type 1
*Mchr1*: MCH receptor 1 gene
MCx: motor cortex
MD: mediodorsal thalamic nucleus
ME: median eminence
MEe: external layer of the median eminence
MEi: internal layer of the median eminence
mfb: medial forebrain bundle
MG: medial geniculate nucleus
mGlu: unspecified metabotropic GLU receptor
mGlu1: metabotropic GLU receptor subtype 1
mGlu5: metabotropic GLU receptor subtype 5
MidThal: midline thalamic nuclei
ml: medial *lemniscus*
MM: medial mammillary nucleus
MnR: median raphe nucleus
MoV: motor nucleus of the V nerve
MPOA: medial preoptic area
MRt: medullary reticular formation
MS: medial septal nucleus
MSN: medium spiny neuron
N/OFQ: nociceptin/orphanin FQ
NCX: Na^+^/Ca^2+^ exchanger
NE: norepinephrine
NEI: neuropeptide E-I
NGE: neuropeptide G-E
NH: neurohypophysis/posterior lobe of the pituitary
NMDAR: NMDA ionotropic GLU receptor
NOP: nociceptin/orphanin Q receptor
NPY: neuropeptide Y
NREM: non-rapid eye movement sleep
NTS: nucleus of the solitary tract
Nts: neurotensin
OB: main olfactory bulb
opt: optic tract
ORX: orexins/hypocretins
ORX_A_: orexin A/hypocretin-1
ORX_B_: orexin B/hypocretin-2
ORXR: unspecified orexin receptor
ORXR_2_: orexin receptor type 2
OT: olfactory tubercle
OXT: oxytocin
OXTR: oxytocin receptor
OVX: ovariectomized
P_4_: progesterone
P2?: unspecified purinergic receptor
PAG: periaqueductal gray matter
PAGvl: ventrolateral periaqueductal gray matter
PAGdm: dorsomedial periaqueductal gray matter
PB: parabrachial nucleus
Pe: periventricular hypothalamic nucleus
PaF: parafascicular thalamic nucleus
PHA: posterior hypothalamic area
PI3K: phosphatidylinositol 3-kinase
PIP3: phosphatidylinositol-3,4,5-trisphosphate
PKA: protein kinase A
PKC: protein kinase C
PLC: phospholipase C
*Pmch*: Pro-melanin-concentrating hormone gene
PnRt: pontine reticular formation
Po: posterior thalamic complex
POMC: proopiomelanocortin
PPTg: pedunculopontine tegmental nucleus
Pr: prepositus nucleus
PreCBL: precerebellar nuclei
Pretect: pretectal nuclei
PV: paraventricular thalamic nucleus
PVH: paraventricular hypothalamic nucleus
PVHp: parvocellular subdivision of the paraventricular hypothalamic nucleus
PVHm: magnocellular subdivision of the paraventricular hypothalamic nucleus
Pir: piriform cortex
REM: rapid eye movement sleep
RIA: radioimmunoassay
rPPTg: rostral pedunculopontine tegmental nucleus
SAL: salivary glands
SuS: superior salivatory nucleus
SC: superior colliculus
scp: superior cerebellar peduncle
SHi: septohippocampal nucleus
SM: supramammillary nucleus
SN: *substantia nigra*
SpCd: spinal cord
SpV: spinal trigeminal nucleus
SSCx: somatosensory cortex
st: *stria terminalis*
SubThal: subthalamus
TRH: thyrotropin-releasing hormone
TRHR: unspecified thyrotropin-releasing hormone receptor
TRPC: transient receptor potential channel
TSH: thyroid-stimulating hormone
UCN1: urocortin 1
UCN2: urocortin 2
UCN3: urocortin 3
V1a: vasopressin receptor subtype 1a
VDCC: voltage-dependent calcium channel
Vest: vestibular nuclei
VL: ventrolateral thalamic nucleus
VMH: ventromedial hypothalamic nucleus
VTA: ventral tegmental area
YR: unspecified NPY receptor
ZI: *zona incerta*
α-MSH: α-melanocyte-stimulating hormone
αR: unspecified adrenergic receptor
α2_A_: adrenergic receptor type 2A
κOR: opioid receptor type κ
